# A Study of Trait Anhedonia in Non-Clinical Chinese Samples: Evidence from the Chapman Scales for Physical and Social Anhedonia

**DOI:** 10.1371/journal.pone.0034275

**Published:** 2012-04-17

**Authors:** Raymond C. K. Chan, Yi Wang, Chao Yan, Qing Zhao, John McGrath, Xiaolu Hsi, William S. Stone

**Affiliations:** 1 Neuropsychology and Applied Cognitive Neuroscience Laboratory, Key Laboratory of Mental Health, Institute of Psychology, Chinese Academy of Sciences, Beijing, China; 2 Graduate School, Chinese Academy of Sciences, Beijing, China; 3 Queensland Centre for Mental Health Research, Park Centre for Mental Health, Wacol, Australia; 4 Queensland Brain Institute, University of Queensland, St Lucia, Australia; 5 Department of Psychiatry, University of Queensland, St Lucia, Australia; 6 MIT Medical, Massachusetts Institute of Technology, Cambridge, Massachusetts, United States of America; 7 Department of Psychiatry, Harvard Medical School, Beth Israel Deaconess Medical Center, Boston, Massachusetts, United States of America; Catholic University of Sacred Heart of Rome, Italy

## Abstract

**Background:**

Recent studies suggest that anhedonia, an inability to experience pleasure, can be measured as an enduring trait in non-clinical samples. In order to examine trait anhedonia in a non-clinical sample, we examined the properties of a range of widely used questionnaires capturing anhedonia.

**Methods:**

887 young adults were recruited from colleges. All of them were administered a set of checklists, including Chapman Scale for Social Anhedonia (CRSAS) and the Chapman Scale for Physical Anhedonia Scale (CPAS), The Temporal Experience of Pleasure Scale(TEPS), and The Schizotypal Personality Questionnaire (SPQ).

**Results:**

Males showed significantly higher level of physical (F = 5.09, p<0.001) and social (F = 4.38, p<0.005) anhedonia than females. As expected, individuals with schizotypal personality features also demonstrated significantly higher scores of physical (t = 3.81, p<0.001) and social (t = 7.33, p<0.001) trait anhedonia than individuals without SPD features, but no difference on self-report anticipatory and consummatory pleasure experience.

**Conclusions:**

Concerning the comparison on each item of physical and social anhedonia, the results indicated that individuals with SPD feature exhibited higher than individuals without SPD features on more items of social anhedonia than physical anhedonia scale. These preliminary findings suggested that trait anhedonia can be identified a non-clinical sample. Exploring the demographic and clinical correlates of trait anhedonia in the general population may provide clues to the pathogenesis of psychotic disorder.

## Introduction

Anhedonia, a key symptom of major depression and schizophrenia, is defined as a reduced capacity to experience pleasure in normally pleasurable situations [1]. Usually, it has been assessed with interview-based rating scales and self-report measures. Chapman and colleagues developed the physical and social anhedonia scales in the late 1970s, namely the Physical Anhedonia Scale (PAS) [2] and the Revised Social Anhedonia Scale (RSAS) [3], to measure stable individual differences in the capacity to experience pleasure from physical-sensual and social-interpersonal sources.

Based on these scales, Fenton and McGlashan found that 76 percent of a schizophrenia sample (n  =  187) showed at least mild anhedonia, and 23 percent showed marked or severe anhedonia as assessed by the SANS [4]. Horan and colleagues reviewed the PAS and the RSAS in schizophrenia patients and controls, in 15 studies. They reported that patients uniformly reported higher levels of both physical and social anhedonia than non-psychiatric controls, which is a pattern that is consistent across symptom states (inpatients vs. outpatients) and during both early and chronic stages of illness [5].

By far, most studies focus on clinical samples of people with schizophrenia or depression, but not on non-clinical sample, although non-clinical samples also experience similar psychotic-like symptoms to lesser degrees (i.e. the “continuum of psychosis”). The median prevalence rate and median incidence rate of psychotic-like delusions and hallucinations is around 5% and 3%, respectively, in the general population [6]. To date, most studies on the continuum of psychosis have focused on positive symptoms rather than negative symptoms. However, schizophrenia is an illness characterized by both positive and negative symptoms. First-onset schizophrenia is associated with a significant degree of negative symptoms [7], and negative symptoms are also present in people with ‘milder’, non-psychotic conditions in the schizophrenia spectrum, such as schizotypal personality disorder (SPD), or people in the general population who show features of ‘schizotypy’ [8]–[11].

Recent research from both animal and imaging studies in humans now indicates that hedonic capacity is not a unitary construct, but can be parsed into two distinct subcomponents, namely consummatory (or liking) and anticipatory (or wanting) pleasure [12]–[15]. Consummatory pleasure refers to the “in the moment” pleasure experienced by an individual directly engaged in an enjoyable activity, whereas anticipatory pleasure refers to the experience of pleasure related to future activities [16]. Based on this construct, Kring and colleagues [16], [17] developed a self-report measure, the Temporal Experience of Pleasure Scale (TEPS), to measure these two components of hedonic capacity in schizophrenia. Recent studies using the TEPS showed evidence for an anticipatory but not a consummatory pleasure deficit in schizophrenia [16].

Prior studies of our lab have focused on studying individuals with SPD features in mainland China, which has led us to adapt and validate several measures and protocols that were developed originally for Western samples. These approaches, which include psychosis proneness, confirmed recently that Chinese samples with SPD features share deficits similar to those seen in Western samples in cognitive functioning, emotional experience and expression, neurological soft signs, and social functioning [18]–[22]. The current study extends this work by focusing on anhedonia in individuals with and without SPD. In particular, it examines qualitative and quantitative differences of anhedonia experienced by a non-clinical sample.

The current study aims to explore the response features of both physical anhedonia and social anhedonia in non-clinical sample of young adults. In addition, we examined trait anhedonia in individuals with and without SPD features using the Chapman Scales for Anhedonia. To date there is a lack of information on the performances of these scales in the Chinese population. As noted above, anhedonia comprises both consummatory and anticipatory components. The current study incorporates this new construct to examine the anticipatory and consummatory pleasure in non-clinical sample in the Chinese setting.

We hypothesized that a non-clinical sample would include individuals with trait anhedonia assessed by Chapman physical and social anhedonia scales, and subjects with SPD features would demonstrate higher levels of physical and social anhedonia than subjects without SPD features.

## Methods

### Ethics Statement

The present study was approved by the ethics committees of the Institute of Psychology. Written informed consent was obtained before the study began. Informed written consent was obtained from all participants and the parents/guardians of the minors who took part in this study before the testing session according to the Declaration of Helsinki.

### Participants

One thousand and thirty-eight university students were recruited from Beijing to complete a set of questionnaires capturing trait anhedonia, schizotypal personality features as well as other measures on pleasure experience. Participants were recruited by announcements before a class by a teacher. Those students who were willing to take part in the study would stay behind the class and completed the questionnaires. They would receive 5RMB in return after completing the questionnaires. Questionnaires were administered in a large group format. The total administration time was about 30 mins. One hundred and sixty-eight of the questionnaires contained missing data, resulting in a final valid sample of 870 participants (369 males and 501 females). The mean ages and education levels of the final sample were 19.24 years (SD = 1.22) and 12.38 years (SD  =  0.49), respectively. According to the manual of the SPQ [23], upper 10% of a standardized high scorers on the SPQ are likely to receive a clinical diagnosis of Schizotypal Personality Disorder, in our sample 92 participants who scored higher than 35 on total score of SPQ were divided into individuals with SPD feature(SPD group). At the same time, we randomly sampled 85 participants of the non-SPD group for more intense scrutiny in the remained sample pool.

### Measures

#### The Revised Physical Anhedonia Scale (PAS) [2]

The Revised Physical Anhedonia Scale (PAS) assesses a self-reported deficit in the ability to experience pleasure from typically pleasurable physical stimuli such as food, sex, and settings. The PAS contains 61 True-False items that yield scores ranging from 0 to 61. High scores indicate more severe physical anhedonia.

#### The Revised Social Anhedonia Scale (RSAS) [3]

The Revised Social Anhedonia Scale (RSAS) assesses deficits in the ability to experience pleasure from non-physical stimuli such as other people, talking or exchanging expressions of feelings. Forty items True-False items constitute the RSAS, and higher score on the RSAS indicates less pleasure from social interactions. Chinese versions of the Revised Social Anhedonia Scale (CRSAS) and the Physical Anhedonia Scale (CPAS) were adopted for this study after undergoing a series of standard validation procedures. First, a panel of experienced psychiatrists, clinical psychologists and neuropsychologists checked the applicability of the items in the Chinese context. The scales then underwent a two-stage translation and back-translation process by two independent translators who majored in psychology. Cronbach’s alpha coefficient was 0.85 for the CRSAS and was 0.86 for the CPAS, in this non-clinical sample, indicating good internal consistency.

#### The Temporal Experience of Pleasure Scale (TEPS) [17]

The Temporal Experience of Pleasure Scale (TEPS) was designed to measure individual trait dispositions in both anticipatory and consummatory experiences of pleasure. It includes a 10-item anticipatory pleasure scale and an 8-item consummatory pleasure scale, which were both reported to be internally consistent and temporally stable. The current study adopted the Chinese version of TEPS [24], which has 20 items capturing four factors: consummatory contextual factor, consummatory abstract factor, anticipatory contextual factor and anticipatory abstract factor. The Chinese version of the TEPS has good clinical discrimination of schizophrenia with negative symptoms from healthy controls.

#### The Schizotypal Personality Questionnaire (SPQ) [23]

The Schizotypal Personality Questionnaire (SPQ) is a self-report scale modeled on the DSM-III-R criteria for schizotypal personality disorder that contains subscales for all nine schizotypal traits. It is used to screen for schizotypal personality disorder in the general population. We adopted the Chinese version of the SPQ [25], which has satisfactory reliability and validity. The reliability of the Chinese version of SPQ was 0.91 for the whole scale and 0.71–0.78 for its three subscales.

### Data Analysis

Descriptive statistics were used to characterize the participants and SPD, non-SPD groups separately. The individual item of physical and social anhedonia scale were examined in males and females respondents separately. Gender effects on positive rates and mean scores were examined by independent sample *t* test.

Based on the Pearson correlations between Chinese version of physical and social anhedonia scales and TEPS, SPQ, we further examined the type responses on physical and social anhedonia, comparison of the mean score of the social and physical anhedonia were conducted between the SPD and non-SPD groups. Moreover, we conducted the *t* test on every items of both physical and social anhedonia scale to find out the differences between SPD and non-SPD groups.

## Results

### Descriptive Analysis and Gender Effect

A sample of 369 male and 501 female (age range 16 to 23) completed the CPAS and CRSAS. The mean physical anhedonia score for males was 16.54 (SD = 8.77), and 13.72 (SD = 6.99) for females, while the mean social anhedonia scale scores for males and females were 9.40 (SD = 6.32) and 7.61 (SD = 5.34) respectively. There was a significant gender effect on both the CPAS (*t* = 5.09, *p*<0.001) and CRSAS scores (*t* = 4.38, *p*<0.001) (Table 1). Males presented higher level of anhedonia than female, both on physical and social anhedonia scale.

**Table 1:** Gender effect on physical and social anhedonia in non-clinical sample.

		N	Mean	SD	*t*	*p*
**CPAS**	Male	369	16.54	8.77	5.09	<0.001
	Female	501	13.72	6.99		
**CRSAS**	Male	369	9.40	6.32	4.38	<0.001
	Female	501	7.61	5.34		

Focusing on respondents for each item of physical and social anhedonia scale, we found that the prevalence of having at least one positive item on the physical and social anhedonia scale for the non-clinical sample ranged from 5.4–89.4% and 4.4–58.2%, respectively.

On the social anhedonia scale, more than 30% participants reported that they have the anhedonia experience on item5 (“*I like to make long distance phone calls to friends and relatives”*, reverse item), item6 (“*Playing with children is a real chore*”), item10 (“*People sometimes think that I am shy when I really just want to be left alone*”), item14 (“*When I am alone, I often resent people telephoning me or knocking on my door*”), item21 (“*People are usually better off if they stay aloof from emotional involvements with most others*”), item22 (“*Although I know I should have affection for certain people, I don’t really feel it*”), item27 (“*I am usually content to just sit alone, thinking and daydreaming*”), item31 (“*I have often found it hard to resist talking to a good friend, even when I have other things to do*”, *reverse item*), item33 (*There are things that are more important to me than privacy*”, *reverse item*) and item37 (“*I find that people too often assume that their daily activities and opinions will be interesting to me*”). Males had higher social anhedonia scores on about half of the RCSAS items. (Details refer to the Appendix S1).

At the same time, on the physical anhedonia scale, more than 40% participants reported that they have the anhedonia experience on item1 (“*I have usually found lovemaking to be intensely pleasurable*”, reverse item), item2 (“When eating a favorite food, I have often tried to eat slowly to make it last longer”, reverse item), item4 (“I have sometimes enjoyed feeling the strength in my muscles”, reverse item), item9 (“I have seldom enjoyed any kind of sexual experience”), item40 (“Sex is okay, but not as much fun as most people claim it is”), item42 (“I have seldom cared to sing in the shower”), item43 (“One food tastes as good as another to me”), item52 (“When I pass by flowers, I have often stopped to smell them”, reverse item) and item53 (“Sex is the most intensely enjoyable thing in life”, reverse item). Males had higher physical anhedonia scores on most of the RCPAS items. Females only had higher anhedonic levels on Item1, 4, 9, 43 and 53. (Details refer to the Appendix S2).

### Correlations between Physical and Social Anhedonia with TEPS and SPQ

Correlation analysis between SPQ, TEPS and CPAS, CRSAS were conducted to examine the validity of anhedonia scales. The SPQ total score was significantly associated with the total scores of the CRSAS and CPAS (r  =  0.45, p<0.001; r  =  0.25, p<0.001, respectively). Although three subscales of the SPQ were significantly correlated with social and physical anhedonia scores, the interpersonal subscale score was most significantly correlated with the CRSAS (r  =  0.57, p<0.001) and the CPAS (r  =  0.38, p<0.001) scores. The correlations between TEPS total score and the CRSAS, CPAS scores were −0.31 (p< 0.001) and −0.52 (p < 0.001), respectively. The four subscales of the TEPS, including abstract anticipatory, contextual anticipatory, abstract consummatory and contextual consummatory, were all significantly inversely associated with the CRSAS and CPAS scores.

### Comparison between Groups with and without SPD Features

**Figure 1:**
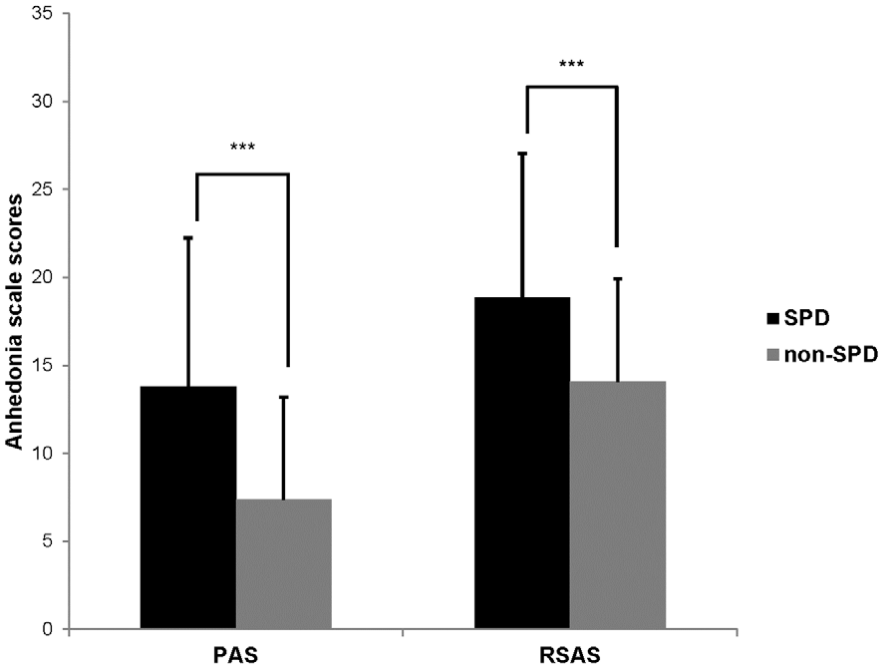
CPAS and CRSAS compared between SPD and non-SPD group. On both physical and social anhedonia scales, SPD group presents higher anhedonic feature than non-SPD group. ^***^ : p< 0.001 (2-tailed).

There were 92 participants in the SPD group and 85 participants in the non-SPD group. There were no significant differences in age or gender between participants with and without SPD features in the non-clinical sample. However, the SPD group produced significantly higher scores on both the physical (t = 3.81, p<0.001) and social (t = 7.33, p<0.001) anhedonia scales than the non-SPD group. As Figure 1 shows, however, the two groups did not differ on the TEPS subscale or total scores.

Further comparison between respondents with and without schizotypal personality on each item of social and physical anhedonia scale, as table 2 and 3 shows, we found that on social anhedonia scale SPD groups presented higher anhedonia level on 26 of 40 items than non-SPD group, however, on physical anhedonia scale, SPD group only presents higher anhedonia level on 18 of 61 items.

**Table 2:** Correlations between RCSAS, CPAS, TEPS and SPQ.

	SPQ cognitive_perceptual	SPQ interpersonal	SPQ disorganized	SPQ total	RCSAS Total	RCPAS Total
**Social Anhedonia total**	0.28***	0.57***	0.34***	0.45***	–	–
**Physical anhedonia total**	0.09^**^	0.38***	0.21***	0.25***	0.52***	–
**TEPS**						
**Abstract_anticipatory**	0.11^**^	−0.20***	−0.01	−0.03	−0.37***	−0.45***
**Contextual_anticipatory**	0.09^**^	−0.13***	−0.02	−0.02	−0.19***	−0.33***
**Abstract_consummatory**	0.06	−0.14***	−0.03	−0.03	−0.29***	−0.53***
**Contextual_consummatory**	0.14***	−0.08^*^	0.05	0.05	−0.17***	−0.33***
**Anticipatory**	0.11^**^	−0.19***	−0.02	−0.03	−0.31***	−0.45***
**Consummatory**	0.11^**^	−0.13***	0.00	0.00	−0.26***	−0.50***
**Total**	0.12***	−0.17***	−0.01	−0.01	−0.31***	−0.52***

*** : p<0.001; **: P<0.01; *: p<0.05.

**Table 3:** SPD and non-SPD group compared on CPAS, CRSAS and TEPS.

	SPD (N = 92)		Non-SPD (N = 85)		*t/χ^2^*	*p*
	Mean	SD	Mean	SD		
**Mean age**	19.39	1.23	19.11	1.29	1.51	0.13
**Gender**	41∶51		34∶51		0.38	0.54
**Education**	12.40	0.49	12.36	0.48	0.51	0.61
**Anhedonia**						
Physical Anhedonia total	18.85	8.19	14.08	8.43	3.81	<0.001
Social Anhedonia total	13.80	5.85	7.38	5.81	7.33	<0.001
**TEPS**						
Abstract Anticipatory	18.89	4.64	19.92	3.42	1.69	0.09
Contextual Anticipatory	17.30	5.36	17.58	4.81	0.35	0.72
Abstract Consummatory	26.96	6.52	27.93	5.12	1.11	0.27
Contextual Consummatory	16.86	4.49	16.31	3.96	−0.87	0.39
Anticipatory pleasure	36.20	8.80	37.49	6.96	−1.09	0.28
Consummatory pleasure	43.82	10.09	44.24	8.15	−0.30	0.76
Total score	80.01	17.75	81.73	13.89	−0.72	0.47

## Discussion

In current study, we examined the trait anhedonia in the Chinese non-clinical sample. The main findings of our study showed that anhedonia did exist in the non-clinical sample ranging from 5.4%–89.4% and 4.4%–58.2% on physical and social anhedonia, respectively. Also, males presented higher level both not only on both the physical and social anhedonia total scores, but also on most of the items of scales. Physical anhedonia and social anhedonia scores were general correlated with the anticipatory and consummatory pleasure, and SPQ scores. Individuals with SPD features exhibited higher physical anhedonia and social anhedonia scores than non-SPD groups, but no differences were found on the anticipatory or consummatory pleasure experiences assessed by TEPS.

Most of these findings are consistent with previous studies on anhedonia in non-clinical sample conducted in western populations. Our results are generally consistent with norms published for (American) Caucasian undergraduate students [26]. For the RSAS score, the mean score for American male college students was 8.91 (SD = 5.12) and for female students was 6.78 (SD = 4.49), males were higher than females with a Cohen’s d of 0.44. For the PAS, the mean score for American male students was 13.71 (SD = 6.80), and for female students was 9.26 (SD = 5.24), males were higher than females with a Cohen’s d of 0.73.

Some empirical studies also found that individuals with proneness to schizophrenia such as SPD had anhedonia experience similar to clinically diagnosed schizophrenia [5], [27], [28]. For example, Horan et al. found that when compared with controls, the “pure” social anhedonics with high scores on the Revised Social Anhedonia Scale had higher levels of schizotypal, schizoid, and paranoid symptoms [28]. Camisa et al. also found that on the social anhedonia scale of psychosis-proneness scales, participants with SPD scored intermediate between non-psychiatric controls and patients with schizophrenia or schizoaffective disorders [29]. A 10-year follow-up study also showed that the demonstration of social anhedonia is predictive of the future development of schizophrenia-spectrum disorders [30]. All these studies suggested that anhedonia could be considered to be traits in both non-clinical and clinical samples. Our current findings suggest that these scales may have utility for the prediction of psychosis in young adults (a question that we plan to explore in future studies).

In our study, Physical and social anhedonia scale scores were positively correlated with SPQ scores, negatively correlated with TEPS scores in the non-clinical sample. Further comparison showed that individuals with SPD features were more physically and socially anhedonic than individuals in the non-SPD group. Previous studies have reported similar results on anhedonia and pleasure experience. For example, Martin et al. found that social anhedonia assessed by Social Anhedonia Scale of Chapman Scales was associated with a decreased attention to positive emotions [31]–[33]. They also examined the relationship between social anhedonia and anticipatory/consummatory pleasure, and found that social anhedonia was associated with both decreased anticipatory and decreased consummatory pleasure. This suggests that social anhedonia may be associated with a general decrease in positive emotions. In our study, there were medium correlations between social anhedonia and TEPS scores (0.17–0.37), physical anhedonia and TEPS scores (0.33–0.52).

As one of the negative symptoms of schizophrenia, anhedonia has been receiving increased attention recently [34]–[36]. A substantial body of evidence has demonstrated the importance of differentiating anticipatory pleasure from consummatory pleasure [1], [37], [38]. Few studies have assessed the anticipatory and consummatory pleasure using TEPS in non-clinical sample and the results were inconsistent. Yan et al. have found that individuals with SPD features demonstrated more problems with self-reported deficits in pleasure experience than those without SPD features, although there was no difference in approach motivation performance [39]. However in our study, we did not find significant difference between these two groups on anticipatory and consummatory pleasure experience. Because of the inconsistent results on anhedonia using different assessments, an integrative systematic assessment needs to be developed to clarify the hedonic capacity deficits in clinical and non-clinical sample. Most recently, an NIMH-funded initiative for the Collaboration to Advance Negative Symptom Assessment in Schizophrenia (CANSAS) had been specifically formed to develop a new negative symptom scale, known as the Clinical Assessment Interview for Negative Symptoms (CAINS) [34]. The CAINS is specifically designed to capture the comprehensive range of negative symptoms in schizophrenia. Anhedonia, in particular, will be examined from the perspective of hedonic capacity vs. performances, anticipatory pleasure vs. consummatory pleasure, and the scope of anhedonia has also been extended to physical, social and recreational/vocational facets. The development of such an interview will help researchers examine anhedonia in schizophrenia spectrum disorders as well as “at-risk mental states” more effectively and comprehensively. Future study need to track high scores over many years to look at rates of other mental health outcomes.

The results of our study should be considered in the context of several limitations. First, this study was not population-based. The findings were obtained from a convenient sample that was recruited from local colleges, thus our results may not generalize to all young adults in the general population. Second, it should be noted that the CRAS and CPAS scores are correlated with the SPQ score. This overlap raises the question of whether high and low CRAS and CPAS scorers were essentially selected for those traits through their SPQ scores. It is a complex and rapidly growing field that needs further extensive research in the near future. Third, the findings on difference between physical anhedonia should be interpreted with caution. Four out of 9 items concerned sex-related issues. One reason that may bias the results may be due to the taboo on sex in Chinese. However, given that the Chinese are now more open about this issue, its effects are likely to be less severe than they were in the past [40], [41]. Moreover, there are also non-sex-related items in the scale. The current findings need to be further examined and replicated in the near future. Finally, our data were limited to self-reported data. As evidence of neurobiological deficits in individuals with SPD and individuals with at-risk mental states accumulates, future studies should incorporate paradigms to examine potential brain structural and functional abnormalities in this sub-clinical sample with high trait anhedonia.

Our findings indicate that a sizable proportion of non-clinical subjects who score high on the CRSAS or the CPAS also experience trait anhedonia. Individuals with SPD features exhibited higher physical anhedonia and social anhedonia scores than non-SPD groups, but no difference on the anticipatory or consummatory pleasure experiences assessed by TEPS. These preliminary findings confirm that trait anhedonia can be identified in a nonclinical sample of young adults. Taken together, this research contributes to our understanding of the continuum of psychosis or risk factors for mental illness.

## Supporting Information

Appendix S1 **Male and Female, SPD and non-SPD respondents score on each item of social anhedonia scale.**
(DOC)

Appendix S2 **Male and Female, SPD and non-SPD respondents score on each item of physical anhedonia scale.**
(DOC)

## References

[1] Harvey PO, Pruessner J, Czechowska Y, Lepage M (2007). Individual differences in trait anhedonia: a structural and functional magnetic resonance imaging study in non-clinical subjects.. Mol Psychiatry.

[2] Chapman LJ, Chapman JP (1978). The Revised Physical Anhedonia Scale, Unpublished test.

[3] Eckblad ML, Chapman LJ, Chapman JP, Mishlove M (1982). The Revised Social Anhedonia Scale,Unpublished test.

[4] Fenton WS, McGlashan TH (1991). Natural History of Schizophrenia Subtypes: II. Positive and Negative Symptoms and Long-term Course.. Arch Gen Psychiatry.

[5] Horan W, Blanchard J, Clark L, Green M (2008). Affective traits in schizophrenia and schizotypy..

[6] van Os J, Linscott RJ, Myin-Germeys I, Delespaul P, Krabbendam L (2009). A systematic review and meta-analysis of the psychosis continuum: evidence for a psychosis proneness?persistence?impairment model of psychotic disorder.. Psychol Med.

[7] Häfner H, Riecher-Rössler A, Maurer K, Fätkenheuer B, Löffler W (1992). First onset and early symptomatology of schizophrenia.. Eur Arch Psychiatry Clin Neurosci.

[8] Faraone SV, Green AI, Seidman LJ, Tsuang MT (2001). “Schizotaxia”: Clinical Implications and New Directions for Research.. Schizophr Bull.

[9] Tsuang MT, Stone WS, Tarbox SI, Faraone SV (2002). An integration of schizophrenia with schizotypy: identification of schizotaxia and implications for research on treatment and prevention.. Schizophr Res.

[10] Meehl PE (1973). Schizotaxia, schizotypy, schizophrenia.. Readings in abnormal psychology.

[11] Meehl PE (1990). Toward an integrated theory of schizotaxia,schizotypy,and schizophrenia.. J Personal Disord.

[12] Berridge KC, Robinson TE (1998). What is the role of dopamine in reward: hedonic impact, reward learning, or incentive salience?. Brain Research Reviews.

[13] Berridge KC, Robinson TE (2003). Parsing reward.. Trends Neurosci.

[14] Hollenbeck JR, Klein HJ (1987). Goal Commitment and the Goal-Setting Process: Problems, Prospects, and Proposals for Future Research.. J Appl Psychol.

[15] Berridge KC, Kringelbach ML (2008). Affective neuroscience of pleasure: reward in humans and animals.. Psychopharmacology (Berl).

[16] Gard D, Kring A, Gard M, Horan W, Green M (2007). Anhedonia in schizophrenia: distinctions between anticipatory and consummatory pleasure.. Schizophr Res.

[17] Gard D, Gard M, Kring A, John O (2006). Anticipatory and consummatory components of the experience of pleasure: a scale development study.. Journal of Research in Personality.

[18] Chan RCK, Li X, Lai M-k, Li H, Wang Y, Cui J (2011). Exploratory study on the base-rate of paranoid ideation in a non-clinical Chinese sample.. Psychiatry Res.

[19] Chan RC, Wang Y, Zhao Q, Yan C, Xu T, Gong QY (2010). Neurological soft signs in individuals with schizotypal personality features.. Aust N Z J Psychiatry.

[20] Chan RCK, Gao X, Li X, Li H, Cui J, Deng Y (2010). The Social Cognition and Interaction Training (SCIT): An extension to individuals with schizotypal personality features.. Psychiatry Res.

[21] Chan RCK, Yan C, Qing Y-H, Wang Y, Wang Y-N, Ma Z (2011). Subjective awareness of everyday dysexecutive behavior precedes ‘objective’ executive problems in schizotypy: A replication and extension study.. Psychiatry Re.

[22] Zong J-g, Chan RCK, Stone WS, Hsi X, Cao X-y, Zhao Q (2010). Coping flexibility in young adults: Comparison between subjects with and without schizotypal personality features.. Schizophr Res.

[23] Raine A (1991). The Spq - a Scale for the Assessment of Schizotypal Personality Based on Dsm-Iii-R Criteria.. Schizophr Bull.

[24] Chan R, Wang Y, Huang J, Shi Y, Hong X, Ma Z (2010). Anticipatory and consummatory components of the experience of pleasure in schizophrenia: cross-cultural validation and extension.. Psychiatry Res.

[25] Chan RCK, Tse WS, Lai MK, Li X, Gao X, Wang Y, et al..

[26] Kwapil TR (1998). Norms on the Wisconsin Psychosis-Proneness Scales for Caucasian undergraduate students in Introductory Psychology courses at the University of Wisconsin-Madison.

[27] Blanchard JJ, Gangestad SW, Brown SA, Horan WP (2000). Hedonic capacity and schizotypy revisited: A taxometric analysis of social anhedonia.. J Abnorm Psychol.

[28] Horan WP, Brown SA, Blanchard JJ (2007). Social anhedonia and schizotypy: The contribution of individual differences in affective traits, stress, and coping.. Psychiatry Res.

[29] Camisa KM, Bockbrader MA, Lysaker P, Rae LL, Brenner CA (2005). Personality traits in schizophrenia and related personality disorders.. Psychiatry Res.

[30] Kwapil TR (1998). Social anhedonia as a predictor of the development of schizophrenia-spectrum disorders.. J Abnorm Psychol.

[31] Berenbaum H, Boden MT, Baker JP, Dizen M, Thompson RJ (2006). Emotional correlates of the different dimensions of schizotypal personality disorder.. J Abnorm Psychol.

[32] Kerns JG (2006). Schizotypy facets, cognitive control, and emotion.. J Abnorm Psychol.

[33] Martin EA, Becker TM, Cicero DC, Docherty AR, Kerns JG (2011). Differential associations between schizotypy facets and emotion traits.. Psychiatry Res.

[34] Blanchard JJ, Kring AM, Horan WP, Gur R (2011). Toward the Next Generation of Negative Symptom Assessments: The Collaboration to Advance Negative Symptom Assessment in Schizophrenia.. Schizophr Bull.

[35] Kirkpatrick B, Strauss GP, Nguyen L, Fischer BA, Daniel DG (2011). The Brief Negative Symptom Scale: Psychometric Properties.. Schizophr Bull.

[36] Marder SR, Daniel DG, Alphs L, Awad AG, Keefe RSE (2011). Methodological Issues in Negative Symptom Trials.. Schizophr Bull.

[37] Auriacombe M, Reneric J, Le Moal M (1997). Animal models of anhedonia.. Psychopharmacology (Berl).

[38] Harvey P-O, Armony J, Malla A, Lepage M (2010). Functional neural substrates of self-reported physical anhedonia in non-clinical individuals and in patients with schizophrenia.. J Psychiatr Res.

[39] Yan C, Liu WH, Cao Y, Chan RC (2011). Self-reported Pleasure Experience and Motivation in Individuals with Schizotypal Personality Disorders Proneness.. East Asian Arch Psychiatry.

[40] Xiao Z, Mehrotra P, Zimmerman R (2011). Sexual revolution in China: implications for Chinese women and society.. AIDS Care.

[41] Zhang K, Beck EJ (1999). Changing sexual attitudes and behaviour in China: Implications for the spread of HIV and other sexually transmitted diseases.. AIDS Care.

