# Supplementary material for: A Study of Trait Anhedonia in Non-Clinical Chinese Samples: Evidence from the Chapman Scales for Physical and Social Anhedonia
Source: PLoS One. 2012 Apr 17;7(4):e34275. doi: 10.1371/journal.pone.0034275 (PMC3328477; doi:10.1371/journal.pone.0034275)
Supplement: Appendix S1 — Male and Female, SPD and non-SPD respondents score on each item of social anhedonia scale. (DOC) [file pone.0034275.s001.doc]

|  | **All Respondents** | | **Males** | | **Females** | |  | **non-SPD** | | **SPD** | |  |
| --- | --- | --- | --- | --- | --- | --- | --- | --- | --- | --- | --- | --- |
|  | **N=870** | | **N=369** | | **N=501** | |  | **n=85** | | **n=92** | |  |
|  | Mean | SD | Mean | SD | Mean | SD | t | Mean | SD | Mean | SD | t |
| RCSAS_01 | 10.69% | 0.31 | 11.65% | 0.32 | 9.98% | 0.30 | 0.79 | 10.59% | 0.31 | 17.39% | 0.38 | -1.31 |
| RCSAS_02 | 4.37% | 0.20 | 6.50% | 0.25 | 2.79% | 0.16 | 2.50** | 1.18% | 0.11 | 9.78% | 0.30 | -2.59* |
| RCSAS_03 | 17.70% | 0.38 | 18.43% | 0.39 | 17.17% | 0.38 | 0.48 | 17.65% | 0.38 | 34.78% | 0.48 | -2.64** |
| RCSAS_04 | 18.16% | 0.39 | 15.99% | 0.37 | 19.76% | 0.40 | 1.44 | 12.94% | 0.34 | 31.52% | 0.47 | -3.05** |
| RCSAS_05 | **30.69%** | **0.46** | 35.77% | 0.48 | 26.95% | 0.44 | 2.77** | 31.76% | 0.47 | 42.39% | 0.50 | -1.47 |
| RCSAS_06 | **32.07%** | **0.47** | 28.18% | 0.45 | 34.93% | 0.48 | -2.13* | 29.41% | 0.46 | 46.74% | 0.50 | -2.40* |
| RCSAS_07 | 24.37% | 0.43 | 27.37% | 0.45 | 22.16% | 0.42 | 1.75 | 23.53% | 0.43 | 23.91% | 0.43 | -0.06 |
| RCSAS_08 | 11.61% | 0.32 | 14.09% | 0.35 | 9.78% | 0.30 | 1.92 | 4.71% | 0.21 | 28.26% | 0.45 | -4.48*** |
| RCSAS_09 | 15.86% | 0.37 | 19.24% | 0.39 | 13.37% | 0.34 | 2.30* | 16.47% | 0.37 | 16.30% | 0.37 | 0.03 |
| RCSAS_10 | **36.67%** | **0.48** | 38.21% | 0.49 | 35.53% | 0.48 | 0.81 | 28.24% | 0.45 | 63.04% | 0.49 | -4.94*** |
| RCSAS_11 | 5.40% | 0.23 | 8.40% | 0.28 | 3.19% | 0.18 | 3.16** | 7.06% | 0.26 | 5.43% | 0.23 | 0.45 |
| RCSAS_12 | 8.51% | 0.28 | 12.20% | 0.33 | 5.79% | 0.23 | 3.37** | 11.76% | 0.32 | 14.13% | 0.35 | -0.47 |
| RCSAS_13 | 16.44% | 0.37 | 18.97% | 0.39 | 14.57% | 0.35 | 1.70 | 9.41% | 0.29 | 41.30% | 0.50 | -5.26*** |
| RCSAS_14 | **33.79%** | **0.47** | 36.31% | 0.48 | 31.94% | 0.47 | 1.34 | 18.82% | 0.39 | 54.35% | 0.50 | -5.27*** |
| RCSAS_15 | 5.40% | 0.23 | 8.67% | 0.28 | 2.99% | 0.17 | 3.69*** | 3.53% | 0.19 | 13.04% | 0.34 | -2.34* |
| RCSAS_16 | 24.48% | 0.43 | 31.44% | 0.46 | 19.36% | 0.40 | 4.03*** | 20.00% | 0.40 | 36.96% | 0.49 | -2.54* |
| RCSAS_17 | 28.05% | 0.45 | 31.98% | 0.47 | 25.15% | 0.43 | 2.20* | 23.53% | 0.43 | 45.65% | 0.50 | -3.17** |
| RCSAS_18 | 20.57% | 0.40 | 22.49% | 0.42 | 19.16% | 0.39 | 1.19 | 22.35% | 0.42 | 38.04% | 0.49 | -2.30* |
| RCSAS_19 | 4.48% | 0.21 | 7.86% | 0.27 | 2.00% | 0.14 | 3.82*** | 7.06% | 0.26 | 11.96% | 0.33 | -1.11 |
| RCSAS_20 | 15.06% | 0.36 | 16.53% | 0.37 | 13.97% | 0.35 | 1.03 | 20.00% | 0.40 | 20.65% | 0.41 | -0.11 |
| RCSAS_21 | **55.40%** | **0.50** | 56.64% | 0.50 | 54.49% | 0.50 | 0.63 | 49.41% | 0.50 | 67.39% | 0.47 | -2.45* |
| RCSAS_22 | **37.24%** | **0.48** | 41.19% | 0.49 | 34.33% | 0.48 | 2.06* | 30.59% | 0.46 | 55.43% | 0.50 | -3.43** |
| RCSAS_23 | 25.52% | 0.44 | 29.54% | 0.46 | 22.55% | 0.42 | 2.31* | 17.65% | 0.38 | 53.26% | 0.50 | -5.33*** |
| RCSAS_24 | 7.01% | 0.26 | 8.94% | 0.29 | 5.59% | 0.23 | 1.86 | 10.59% | 0.31 | 17.39% | 0.38 | -1.31 |
| RCSAS_25 | 7.24% | 0.26 | 11.11% | 0.31 | 4.39% | 0.21 | 3.58*** | 7.06% | 0.26 | 17.39% | 0.38 | -2.13* |
| RCSAS_26 | 9.08% | 0.29 | 12.20% | 0.33 | 6.79% | 0.25 | 2.65** | 7.06% | 0.26 | 20.65% | 0.41 | -2.68** |
| RCSAS_27 | **37.59%** | **0.48** | 37.94% | 0.49 | 37.33% | 0.48 | 0.19 | 30.59% | 0.46 | 70.65% | 0.46 | -5.78*** |
| RCSAS_28 | 14.60% | 0.35 | 18.70% | 0.39 | 11.58% | 0.32 | 2.87** | 10.59% | 0.31 | 45.65% | 0.50 | -5.65*** |
| RCSAS_29 | 24.14% | 0.43 | 27.37% | 0.45 | 21.76% | 0.41 | 1.89 | 21.18% | 0.41 | 47.83% | 0.50 | -3.88*** |
| RCSAS_30 | 27.36% | 0.45 | 29.54% | 0.46 | 25.75% | 0.44 | 1.23 | 21.18% | 0.41 | 26.09% | 0.44 | -0.76 |
| RCSAS_31 | **58.16%** | **0.49** | 58.54% | 0.49 | 57.88% | 0.49 | 0.19 | 56.47% | 0.50 | 54.35% | 0.50 | 0.28 |
| RCSAS_32 | 7.70% | 0.27 | 11.38% | 0.32 | 4.99% | 0.22 | 3.33** | 8.24% | 0.28 | 18.48% | 0.39 | -2.03* |
| RCSAS_33 | **34.14%** | **0.47** | 34.42% | 0.48 | 33.93% | 0.47 | 0.15 | 38.82% | 0.49 | 27.17% | 0.45 | 1.65 |
| RCSAS_34 | 15.98% | 0.37 | 18.70% | 0.39 | 13.97% | 0.35 | 1.85 | 11.76% | 0.32 | 36.96% | 0.49 | -4.09*** |
| RCSAS_35 | 29.77% | 0.46 | 34.96% | 0.48 | 25.95% | 0.44 | 2.85** | 24.71% | 0.43 | 59.78% | 0.49 | -5.03*** |
| RCSAS_36 | 14.02% | 0.35 | 16.53% | 0.37 | 12.18% | 0.33 | 1.80 | 16.47% | 0.37 | 28.26% | 0.45 | -1.90 |
| RCSAS_37 | **31.03%** | **0.46** | 36.04% | 0.48 | 27.35% | 0.45 | 2.72** | 21.18% | 0.41 | 54.35% | 0.50 | -4.83*** |
| RCSAS_38 | 16.21% | 0.37 | 18.70% | 0.39 | 14.37% | 0.35 | 1.69 | 18.82% | 0.39 | 39.13% | 0.49 | -3.05** |
| RCSAS_39 | 7.70% | 0.27 | 12.74% | 0.33 | 3.99% | 0.20 | 4.49*** | 9.41% | 0.29 | 18.48% | 0.39 | -1.76 |
| RCSAS_40 | 12.76% | 0.33 | 14.09% | 0.35 | 11.78% | 0.32 | 1.00 | 10.59% | 0.31 | 31.52% | 0.47 | -3.54** |

***. Correlation is significant at the 0.001 level (2-tailed); **. Correlation is significant at the 0.01 level (2-tailed); *. Correlation is significant at the 0.05 level (2-tailed).
