# Supplementary material for: A Study of Trait Anhedonia in Non-Clinical Chinese Samples: Evidence from the Chapman Scales for Physical and Social Anhedonia
Source: PLoS One. 2012 Apr 17;7(4):e34275. doi: 10.1371/journal.pone.0034275 (PMC3328477; doi:10.1371/journal.pone.0034275)
Supplement: Appendix S2 — Male and Female, SPD and non-SPD respondents score on each item of physical anhedonia scale. (DOC) [file pone.0034275.s002.doc]

|  | **All Respondents** | | **Males** | | **Females** | |  | **non-SPD** | | **SPD** | |  |
| --- | --- | --- | --- | --- | --- | --- | --- | --- | --- | --- | --- | --- |
|  | **N=870** | | **N=369** | | **N=501** | |  | **n=85** | | **n=92** | |  |
|  | Mean | SD | Mean | SD | Mean | SD | t | Mean | SD | Mean | SD | t |
| RCPAS_01 | **47.47%** | 0.50 | 22.22% | 0.42 | 66.07% | 0.47 | -14.47*** | 48.24% | 0.50 | 46.74% | 0.50 | 0.20 |
| RCPAS_02 | **43.68%** | 0.50 | 40.38% | 0.49 | 46.11% | 0.50 | -1.69 | 41.18% | 0.50 | 42.39% | 0.50 | -0.16 |
| RCPAS_03 | 22.76% | 0.42 | 28.18% | 0.45 | 18.76% | 0.39 | 3.22** | 20.00% | 0.40 | 23.91% | 0.43 | -0.63 |
| RCPAS_04 | **60.46%** | 0.49 | 32.25% | 0.47 | 81.24% | 0.39 | -16.34*** | 61.18% | 0.49 | 53.26% | 0.50 | 1.06 |
| RCPAS_05 | 25.52% | 0.44 | 36.31% | 0.48 | 17.56% | 0.38 | 6.19*** | 23.53% | 0.43 | 23.91% | 0.43 | -0.06 |
| RCPAS_06 | 20.57% | 0.40 | 26.02% | 0.44 | 16.57% | 0.37 | 3.34** | 18.82% | 0.39 | 27.17% | 0.45 | -1.32 |
| RCPAS_07 | 25.17% | 0.43 | 33.06% | 0.47 | 19.36% | 0.40 | 4.53*** | 23.53% | 0.43 | 30.43% | 0.46 | -1.03 |
| RCPAS_08 | 11.95% | 0.32 | 14.36% | 0.35 | 10.18% | 0.30 | 1.84 | 9.41% | 0.29 | 26.09% | 0.44 | -2.98** |
| RCPAS_09 | **48.16%** | 0.50 | 44.17% | 0.50 | 51.10% | 0.50 | -2.02* | 37.65% | 0.49 | 60.87% | 0.49 | -3.16** |
| RCPAS_10 | 26.32% | 0.44 | 31.44% | 0.46 | 22.55% | 0.42 | 2.90** | 20.00% | 0.40 | 39.13% | 0.49 | -2.85** |
| RCPAS_11 | 16.32% | 0.37 | 20.87% | 0.41 | 12.97% | 0.34 | 3.04** | 16.47% | 0.37 | 28.26% | 0.45 | -1.90 |
| RCPAS_12 | 17.24% | 0.38 | 23.85% | 0.43 | 12.38% | 0.33 | 4.31*** | 24.71% | 0.43 | 28.26% | 0.45 | -0.53 |
| RCPAS_13 | 13.10% | 0.34 | 18.70% | 0.39 | 8.98% | 0.29 | 4.05*** | 12.94% | 0.34 | 22.83% | 0.42 | -1.73 |
| RCPAS_14 | 23.68% | 0.43 | 28.46% | 0.45 | 20.16% | 0.40 | 2.80** | 24.71% | 0.43 | 31.52% | 0.47 | -1.01 |
| RCPAS_15 | 17.01% | 0.38 | 18.70% | 0.39 | 15.77% | 0.36 | 1.13 | 18.82% | 0.39 | 36.96% | 0.49 | -2.74** |
| RCPAS_16 | 13.91% | 0.35 | 17.89% | 0.38 | 10.98% | 0.31 | 2.83** | 14.12% | 0.35 | 26.09% | 0.44 | -2.01* |
| RCPAS_17 | 18.05% | 0.38 | 25.20% | 0.43 | 12.77% | 0.33 | 4.58*** | 16.47% | 0.37 | 30.43% | 0.46 | -2.22* |
| RCPAS_18 | 36.21% | 0.48 | 35.77% | 0.48 | 36.53% | 0.48 | -0.23 | 32.94% | 0.47 | 39.13% | 0.49 | -0.85 |
| RCPAS_19 | 11.26% | 0.32 | 16.80% | 0.37 | 7.19% | 0.26 | 4.25*** | 9.41% | 0.29 | 23.91% | 0.43 | -2.64** |
| RCPAS_20 | 26.67% | 0.44 | 29.27% | 0.46 | 24.75% | 0.43 | 1.48 | 18.82% | 0.39 | 31.52% | 0.47 | -1.96 |
| RCPAS_21 | 12.30% | 0.33 | 17.07% | 0.38 | 8.78% | 0.28 | 3.55*** | 9.41% | 0.29 | 26.09% | 0.44 | -2.98** |
| RCPAS_22 | 36.32% | 0.48 | 40.38% | 0.49 | 33.33% | 0.47 | 2.13* | 30.59% | 0.46 | 48.91% | 0.50 | -2.52* |
| RCPAS_23 | 35.98% | 0.48 | 34.96% | 0.48 | 36.73% | 0.48 | -0.54 | 34.12% | 0.48 | 40.22% | 0.49 | -0.84 |
| RCPAS_24 | 17.36% | 0.38 | 26.56% | 0.44 | 10.58% | 0.31 | 5.96*** | 15.29% | 0.36 | 25.00% | 0.44 | -1.62 |
| RCPAS_25 | 10.46% | 0.31 | 14.63% | 0.35 | 7.39% | 0.26 | 3.32** | 10.59% | 0.31 | 15.22% | 0.36 | -0.91 |
| RCPAS_26 | 17.36% | 0.38 | 19.78% | 0.40 | 15.57% | 0.36 | 1.60 | 17.65% | 0.38 | 28.26% | 0.45 | -1.69 |
| RCPAS_27 | 34.14% | 0.47 | 34.69% | 0.48 | 33.73% | 0.47 | 0.29 | 29.41% | 0.46 | 39.13% | 0.49 | -1.36 |
| RCPAS_28 | 7.70% | 0.27 | 12.74% | 0.33 | 3.99% | 0.20 | 4.49*** | 9.41% | 0.29 | 13.04% | 0.34 | -0.76 |
| RCPAS_29 | 17.47% | 0.38 | 25.47% | 0.44 | 11.58% | 0.32 | 5.18*** | 16.47% | 0.37 | 23.91% | 0.43 | -1.23 |
| RCPAS_30 | 20.80% | 0.41 | 30.35% | 0.46 | 13.77% | 0.34 | 5.82*** | 22.35% | 0.42 | 20.65% | 0.41 | 0.27 |
| RCPAS_31 | 14.25% | 0.35 | 17.89% | 0.38 | 11.58% | 0.32 | 2.57* | 11.76% | 0.32 | 33.70% | 0.48 | -3.61*** |
| RCPAS_32 | 22.76% | 0.42 | 25.20% | 0.43 | 20.96% | 0.41 | 1.46 | 23.53% | 0.43 | 25.00% | 0.44 | -0.23 |
| RCPAS_33 | 12.53% | 0.33 | 15.99% | 0.37 | 9.98% | 0.30 | 2.58* | 12.94% | 0.34 | 14.13% | 0.35 | -0.23 |
| RCPAS_34 | 10.92% | 0.31 | 14.63% | 0.35 | 8.18% | 0.27 | 2.92** | 12.94% | 0.34 | 16.30% | 0.37 | -0.63 |
| RCPAS_35 | 24.94% | 0.43 | 21.95% | 0.41 | 27.15% | 0.45 | -1.77 | 30.59% | 0.46 | 32.61% | 0.47 | -0.29 |
| RCPAS_36 | 27.24% | 0.45 | 26.02% | 0.44 | 28.14% | 0.45 | -0.70 | 20.00% | 0.40 | 31.52% | 0.47 | -1.76 |
| RCPAS_37 | 24.94% | 0.43 | 29.00% | 0.45 | 21.96% | 0.41 | 2.34* | 27.06% | 0.45 | 31.52% | 0.47 | -0.65 |
| RCPAS_38 | 5.40% | 0.23 | 9.49% | 0.29 | 2.40% | 0.15 | 4.24*** | 3.53% | 0.19 | 13.04% | 0.34 | -2.34* |
| RCPAS_39 | 11.38% | 0.32 | 15.18% | 0.36 | 8.58% | 0.28 | 2.93** | 5.88% | 0.24 | 17.39% | 0.38 | -2.43* |
| RCPAS_40 | **45.98%** | 0.50 | 43.90% | 0.50 | 47.50% | 0.50 | -1.05 | 40.00% | 0.49 | 58.70% | 0.50 | -2.52* |
| RCPAS_41 | 39.54% | 0.49 | 47.15% | 0.50 | 33.93% | 0.47 | 3.94*** | 35.29% | 0.48 | 39.13% | 0.49 | -0.53 |
| RCPAS_42 | **43.45%** | 0.50 | 43.90% | 0.50 | 43.11% | 0.50 | 0.23 | 44.71% | 0.50 | 52.17% | 0.50 | -0.99 |
| RCPAS_43 | **89.54%** | 0.31 | 86.18% | 0.35 | 92.02% | 0.27 | -2.69** | 89.41% | 0.31 | 86.96% | 0.34 | 0.50 |
| RCPAS_44 | 22.64% | 0.42 | 33.88% | 0.47 | 14.37% | 0.35 | 6.67*** | 25.88% | 0.44 | 21.74% | 0.41 | 0.64 |
| RCPAS_45 | 9.54% | 0.29 | 13.82% | 0.35 | 6.39% | 0.24 | 3.53*** | 8.24% | 0.28 | 15.22% | 0.36 | -1.45 |
| RCPAS_46 | 22.18% | 0.42 | 27.10% | 0.45 | 18.56% | 0.39 | 2.95** | 24.71% | 0.43 | 22.83% | 0.42 | 0.29 |
| RCPAS_47 | 24.60% | 0.43 | 26.29% | 0.44 | 23.35% | 0.42 | 0.99 | 25.88% | 0.44 | 35.87% | 0.48 | -1.44 |
| RCPAS_48 | 22.87% | 0.42 | 31.44% | 0.46 | 16.57% | 0.37 | 5.06*** | 9.41% | 0.29 | 23.91% | 0.43 | -2.64** |
| RCPAS_49 | 9.66% | 0.30 | 12.47% | 0.33 | 7.58% | 0.27 | 2.34* | 10.59% | 0.31 | 14.13% | 0.35 | -0.71 |
| RCPAS_50 | 24.94% | 0.43 | 30.08% | 0.46 | 21.16% | 0.41 | 2.97** | 21.18% | 0.41 | 33.70% | 0.48 | -1.88 |
| RCPAS_51 | 8.85% | 0.28 | 13.55% | 0.34 | 5.39% | 0.23 | 3.98*** | 4.71% | 0.21 | 22.83% | 0.42 | -3.65*** |
| RCPAS_52 | **40.80%** | 0.49 | 47.70% | 0.50 | 35.73% | 0.48 | 3.55*** | 40.00% | 0.49 | 38.04% | 0.49 | 0.27 |
| RCPAS_53 | **69.54%** | 0.46 | 55.01% | 0.50 | 80.24% | 0.40 | -8.02*** | 72.94% | 0.45 | 64.13% | 0.48 | 1.26 |
| RCPAS_54 | 9.89% | 0.30 | 12.20% | 0.33 | 8.18% | 0.27 | 1.91 | 12.94% | 0.34 | 16.30% | 0.37 | -0.63 |
| RCPAS_55 | 20.34% | 0.40 | 22.49% | 0.42 | 18.76% | 0.39 | 1.34 | 18.82% | 0.39 | 30.43% | 0.46 | -1.80 |
| RCPAS_56 | 14.02% | 0.35 | 18.70% | 0.39 | 10.58% | 0.31 | 3.31** | 7.06% | 0.26 | 27.17% | 0.45 | -3.70*** |
| RCPAS_57 | 13.68% | 0.34 | 17.07% | 0.38 | 11.18% | 0.32 | 2.44** | 10.59% | 0.31 | 23.91% | 0.43 | -2.38* |
| RCPAS_58 | 15.06% | 0.36 | 19.51% | 0.40 | 11.78% | 0.32 | 3.07** | 17.65% | 0.38 | 25.00% | 0.44 | -1.19 |
| RCPAS_59 | 15.40% | 0.36 | 22.49% | 0.42 | 10.18% | 0.30 | 4.81*** | 14.12% | 0.35 | 13.04% | 0.34 | 0.21 |
| RCPAS_60 | 6.44% | 0.25 | 11.38% | 0.32 | 2.79% | 0.16 | 4.74*** | 3.53% | 0.19 | 11.96% | 0.33 | -2.13* |
| RCPAS_61 | 32.87% | 0.47 | 41.46% | 0.49 | 26.55% | 0.44 | 4.60*** | 34.12% | 0.48 | 39.13% | 0.49 | -0.69 |

***. Correlation is significant at the 0.001 level (2-tailed); **. Correlation is significant at the 0.01 level (2-tailed); *. Correlation is significant at the 0.05 level (2-tailed).
